# Association between Hoehn and Yahr, Mini-Mental State Examination, age, and clinical syndrome predominance and diagnostic effectiveness of ioflupane I 123 injection (DaTSCAN™) in subjects with clinically uncertain parkinsonian syndromes

**DOI:** 10.1186/s13195-014-0067-0

**Published:** 2014-10-08

**Authors:** Nin Bajaj, Robert A Hauser, John Seibyl, Andreas Kupsch, Michail Plotkin, Chris Chen, Igor D Grachev

**Affiliations:** 1 National Parkinson’s Foundation of Excellence, Nottingham University Hospitals NHS Trust and University of Nottingham, Derby Road, Nottingham NG7 2UH, UK; 2 USF Parkinson’s Disease and Movement Disorders Center, National Parkinson Foundation Center of Excellence, USF Health - Byrd Institute, 4001 E. Fletcher Ave, 6th Floor, Tampa 33613, FL, USA; 3 The Institute for Neurodegenerative Disorders, 60 Temple St, New Haven 06510, CT, USA; 4 Otto-von-Guericke-University, Leipziger Strasse 44, Magdeburg, 39120, Germany; 5 Leiter des Vivantes Instituts für Nuklearmedizin Mitte/Nord, Landsberger Allee 49, Berlin, 10249, Germany; 6 H2O Clinical LLC, 200 International Circle, Suite 5888, Cockeysville 21030, MD, USA; 7 Medical Affairs, GE Healthcare – Life Sciences, 101 Carnegie Center, Princeton NJ 08540, New Jersey, USA; 8 Presently: Novartis Consumer Health, Parsippany, New Jersey, USA; 9 Presently: Genpact Pharmalink, Short Hills, New Jersey, USA

## Abstract

**Introduction:**

Diagnostic effectiveness of Ioflupane I 123 injection (DaTSCAN™, DaTscan™, or [123I]FP-CIT or ioflupane [^123^I]) SPECT imaging, was assessed in patients with clinically uncertain parkinsonian syndrome (CUPS).

**Methods:**

We investigated the association between subject’s Hoehn & Yahr (H&Y) stage, Mini-Mental State Examination (MMSE), age, and motor symptom subgroups and diagnostic performance of ioflupane [^123^I] imaging. Phase 4 study data were used to calculate sensitivity, specificity, positive and negative predictive value, and accuracy in 92 CUPS subjects, using 1-year clinical diagnosis after ioflupane [^123^I] imaging as reference standard.

**Results:**

Diagnostic effectiveness of ioflupane [^123^I] imaging was high in all subgroups: 91% to 100% for H&Y low (<2) and high (≥2) stage subjects; 93% to 96% for MMSE low (<29) or high (≥29) scores; 91% to100% in both age subgroups (younger [<68] and older [≥68]); and 92% to 100% in subjects with both tremor dominant and balanced motor signs. Specificity of ioflupane [^123^I] imaging for bradykinetic rigid or posturally (BRP) unstable motor subtype was lower, but better than for baseline clinical diagnosis.

**Conclusions:**

Strongest diagnostic performance of ioflupane [^123^I] imaging for clinical diagnosis of Parkinson’s syndrome (PS) or non-PS was associated with tremor and balanced motor dominance rather than with BRP dominance. High diagnostic effectiveness of ioflupane [^123^I] imaging and favourable performance relative to final clinical diagnosis at 1 year post-scan in subjects with CUPS was demonstrated. This study suggests that the diagnostic performance of ioflupane [^123^I] imaging in CUPS remains high at all stages of disease, including early stage, and across both age groups and cognitive state (MMSE).

## 1 Introduction

The early clinical diagnosis of parkinsonian syndromes (PS) is made difficult by subtle and nonspecific presenting symptoms. A randomized study of early, clinically uncertain parkinsonian syndrome (CUPS) showed that the availability of Ioflupane I 123 injection (DaTSCAN™ or DaTscan™ or [^123^I]FP-CIT or ioflupane [^123^I]) imaging results often led to a significant change in diagnosis from baseline to 1 year (observed in 55/102 (54%) subjects who received ioflupane [^123^I] imaging vs. 26/113 (23%) controls who did not receive ioflupane [^123^I] imaging), an increase in physician’s confidence of the revised diagnosis, and a significant change in clinical management [1]. In the current study, we investigated the effect of subject’s age, disease stage, and other clinical and neurocognitive measures on diagnostic performance of ioflupane [^123^I] imaging that have not been reported previously. We report the diagnostic effectiveness (sensitivity, specificity, positive predictive value (PPV), negative predictive value (NPV), and accuracy) of ioflupane [^123^I] imaging versus the final clinical diagnosis for subjects who had both ioflupane [^123^I] imaging results at baseline and a specific clinical diagnosis 1 year post scan, including analyses based on subject’s Hoehn and Yahr (H&Y) stage, Mini-Mental State Examination (MMSE) score, age, and predominant clinical syndrome subgroup. It has already been established that age is a contributing factor in the progressive decline of dopamine transporter (DaT) binding in healthy aging subjects [2]. However, it has also been shown that age of Parkinson’s disease (PD) patients contributes to disease severity, independent of the duration of disease [3].

Possible effects of age and gender on disease severity were investigated by Szewczyk-Krolikowski and colleagues, who observed some clinical/phenotypic heterogeneity in age and gender subgroups, but the study did not include ioflupane [^123^I] imaging [3]. Other investigators have attempted to use changes in motor symptom scales (Unified Parkinson’s Disease Rating Scale) as predictors of clinical course, but found no change over time in a small group of patients with scans without evidence for dopaminergic deficiency with DaT single-photon emission computed tomography (SPECT) [4]. Whilst the Unified Parkinson’s Disease Rating Scale effectively measures the progression of PD symptoms for the first 10 years of the disease [4], other investigators have used the H&Y stage, as in the current study, rather than the Unified Parkinson’s Disease Rating Scale [5]. Nissen and colleagues found an inverse correlation of dopamine transporter uptake ratios with H&Y stage and dosage of antiparkinson drugs [5].

The current analysis was undertaken to evaluate whether particular patient characteristics – specifically H&Y stage, MMSE score, age, and clinical syndrome phenotype – were associated with relatively better or worse diagnostic performance of ioflupane [^123^I] imaging in CUPS using 1-year post-scan clinical diagnosis as a reference standard.

## 2 Methods

The clinical and imaging methods were reported previously [1]. Ioflupane [^123^I] has been validated in several clinical trials and was approved for use in 2000 in the EU and in 2011 in the USA [6],[7]. Of the 122 subjects from the phase 4 clinical trial who received ioflupane [^123^I] imaging studies, 30 subjects had nonconfirmed diagnoses at 1 year, disqualifying them from being included as a reference standard (confirmed diagnosis at 1 year), resulting in 92 subjects with CUPS who were qualified for this analysis [1]. The clinical trial was approved by local ethics committees and their institutional review boards (see Additional file 1) and was conducted under the Declaration of Helsinki. Informed consent was obtained by subjects or their guardians for that trial, which covered data analysis and publications related to the study. Ninety-two subjects with complete subgrouping datasets (baseline H&Y stage, baseline MMSE score, baseline age, predominant motor syndrome, ioflupane [^123^I] imaging results. and 1-year clinical diagnosis) were included in this report. To analyze the effect of the H&Y stage on diagnostic effectiveness of ioflupane [^123^I] imaging, subjects were classified using a baseline-modified H&Y stage dichotomized into two groups: stage <2 and stage ≥2. To analyze the effect of cognitive status of subjects on diagnostic effectiveness of ioflupane [^123^I] imaging, subjects were grouped by scores <29 and ≥29 (for a detailed explanation of this methodological approach, see Statistical methods). To analyze the effect of subject’s age on the diagnostic effectiveness of ioflupane [^123^I] imaging, subjects were classified as <68 or ≥68 years of age at baseline. Investigators also grouped subjects at baseline into a predominant clinical motor syndrome subgroup – tremor dominant; bradykinesia, rigidity, postural instability (BRP) dominant; or balanced (regarding tremor and bradykinesia scores) – to determine the effect of motor symptom subtype on diagnostic effectiveness of ioflupane [^123^I] imaging.

Subjects in the tremor dominant subgroup had tremor scores greater than their maximal score for bradykinesia, rigidity, or postural instability. Subjects in the BRP dominant subgroup had the maximal score of bradykinesia, rigidity, or postural instability greater than their score for tremor. Subjects in the balanced group had maximal BRP scores equal to tremor scores. The scores were calculated by assigning numbers scores for motor signs: 1 for none, 2 for possible, and 3 for definite.

### 2.1 Data source

Data analyzed included baseline H&Y stage, MMSE score, age at baseline, predominant clinical syndrome subgroup, and ioflupane [^123^I] imaging results in subjects with CUPS [1]. Subjects with CUPS defined as monosymptomatic, atypical or incomplete presentation of tremor, rigidity, bradykinesia or postural instability, and/or had a poor response to levodopa were enrolled in the study. Please see Methods for the selection process of the 92 subjects from 19 hospital centers in Europe (15 centers) and the USA (four centers) who were included in this report. Subjects in this study had experienced motor and nonmotor signs and symptoms, and were under physician observation for up to a maximum of 5 years prior to ioflupane [^123^I] imaging, which in combination with 1-year post-scan clinical follow-up provided sufficient rationale for using the 1-year clinical diagnosis as a reference standard (standard of truth). The study population receiving ioflupane [^123^I] imaging from which the 92 subjects were selected to be analyzed had been symptomatic for 2.54 years (mean) or 2.20 years (median) [1]. Subjects with clinically diagnosed vascular lesions were not intentionally enrolled, although enrolling older subjects with some silent vascular changes would have been unavoidable.

### 2.2 Interpretation of Ioflupane [^123^I] imaging results

Description of the ioflupane [^123^I] SPECT imaging methodology has been reported previously [1]. Each reconstructed ioflupane [^123^I] SPECT image was categorized as normal or abnormal by an onsite imaging reader (a nuclear medicine physician having expertise in neuroimaging) and interpreted with no access to clinical information, such as symptoms, clinical signs evolution, treatment, and clinical management changes.

### 2.3 Statistical methods

The statistical analysis plan was to generate diagnostic efficacy parameters – sensitivity (equivalent to positive percent agreement), specificity (equivalent to negative percent agreement), PPV, NPV, and accuracy – for ioflupane [^123^I] imaging in subject subgroups. The subgroups for H&Y stage, MMSE, and subject’s age were based on dichotomization; that is, an entire population at the median values (to ensure the same number of subjects in low and high subgroups). Dichotomization is a common statistical approach used with limited datasets for which categorical, more meaningful subdivisions requiring a much larger sample size cannot be used. The median values were chosen as the cutoff points to avoid subjectivity by the authors and to prevent outliers from having undue influence. The values we used were H&Y median score of 2, MMSE median score of 29, and median age of 68 years, respectively. The subgroups for dominant motor syndrome were based on a clinically common classification into tremor dominant, BRP dominant, and balanced subgroups. Standard methods were employed to calculate the diagnostic efficacy parameters for ioflupane [^123^I] imaging using 1-year post-scan clinical diagnosis as the reference standard (standard of truth). For subjects with a PS diagnosis at 1 year, an ioflupane [^123^I] image reading of abnormal was deemed a true positive, whilst an ioflupane [^123^I] reading of normal was deemed a false negative. For subjects with a non-PS diagnosis at 1 year, an ioflupane [^123^I] image reading of normal was deemed a true negative, whereas an ioflupane [^123^I] image reading of abnormal was deemed a false positive. Comparison of diagnostic efficacy parameters between mutually exclusive subgroups was performed with Fisher’s exact test.

## 3 Results

### 3.1 Study population

The number of subjects analyzed in four subgroups was 92, with approximately equal distribution between normal and abnormal ioflupane [^123^I] imaging results among all subgroups. Table 1 summarizes the various subject subgroups in relation to the 1-year clinical diagnosis and ioflupane [^123^I] imaging results.

**Table 1:** **Subject subgroup relationship to 1-year clinical diagnosis (standard of truth) and ioflupane [**^**123**^**I] imaging results (*n*** **= 92)**

**Subjects with CUPS**		**Subgroup: H&Y stage**			**Subgroup: MMSE score**			**Subgroup: age of subjects at baseline**			**Subgroup: clinical syndrome predominance at baseline**^**a**^			
**1-year clinical diagnosis**	**Ioflupane [**^**123**^**I] imaging results**	**<2**	**≥2**	**Total**	**<29**	**≥29**	**Total**	**<68 years**	**≥68 years**	**Total**	**TD**	**BRP dominant**	**B**	**Total**
**PS**	Normal	2	1	3	1	2	3	2	1	3	0	2	1	3
	Abnormal	26	20	46	19	27	46	15	31	46	10	17	19	46
**Non-PS**	Normal	20	21	41	16	25	41	15	26	41	25	4	12	41
	Abnormal	0	2	2	1	1	2	2	0	2	0	2	0	2
**Total**		48	44	92	37	55	92	34	58	92	35	25	32	92

Data presented as numbers. B, balanced; BRP, bradykinesia, rigidity, postural instability; CUPS, clinically uncertain parkinsonian syndrome; H&Y, Hoehn and Yahr; MMSE, Mini-Mental State Examination; PS, parkinsonian syndrome; TD, tremor dominant. ^a^TD, tremor score > BRP; BRP dominant, BRP > tremor score; B, maximal score of BRP = tremor score. Scores for these motor signs were 1 for none, 2 for possible, and 3 for definite.

### 3.2 Hoehn and Yahr stage

At baseline, most of the 92 subjects in this group were in the early stages of disease (H&Y stages 1 to 2), split about evenly between the two stages. Slightly less than one-half of the subjects had normal ioflupane [^123^I] images. The subjects who had abnormal ioflupane [^123^I] images were about evenly split between H&Y stages 1 and 2.

**Figure 1:**
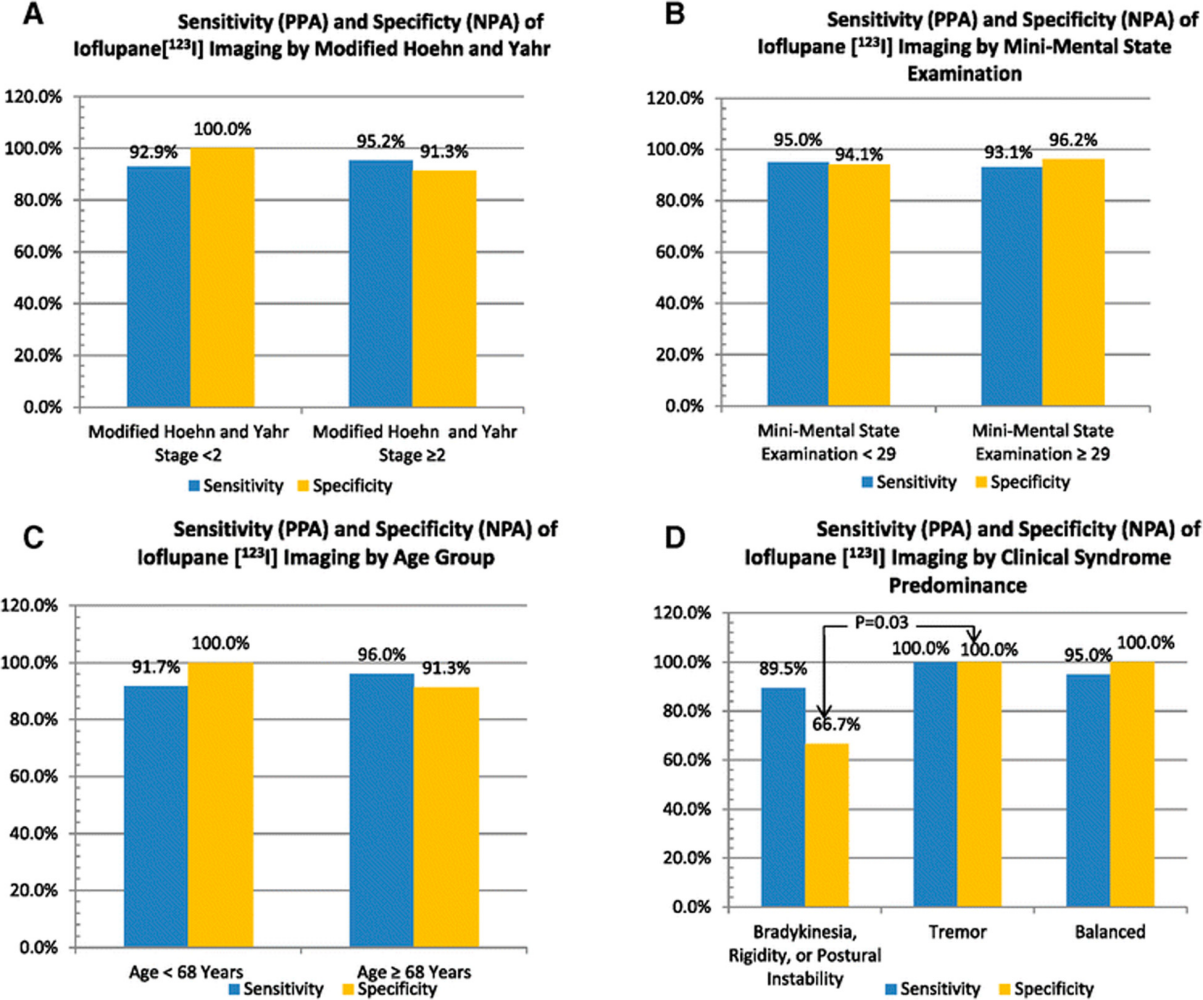
**Sensitivity and specificity of ioflupane [**^**123**^**I] imaging for prediction of clinical diagnosis, 1 year. (A)** Modified Hoehn and Yahr Stage. **(B)** Mini-Mental State Examination Score. **(C)** Age of subjects. **(D)** Clinical syndrome predominance. **NPA,** negative percent agreement; **PPA,** positive percent agreement.

Efficacy parameters for ioflupane [^123^I] imaging ranged between 91 and 100% to determine whether or not subjects had PS or non-PS at 1 year for all diagnostic efficacy parameters: sensitivity, specificity, accuracy, PPV, and NPV. In addition, all of the efficacy parameters showed similar high values for subject groups having either low (<2) or high (≥2) H&Y stage scores (Table 2, Figure 1A).

**Table 2:** **Efficacy parameters for prediction of clinical diagnosis 1 year post scan: modified H&Y stage (*n*** **= 92)**

**Subjects with CUPS**	**Clinical diagnosis at 1 year post-scan**					
	**Subgroup: modified H&Y stage <2**			**Subgroup: modified H&Y stage ≥2**		
	**PS**	**Non-PS**	**Total**	**PS**	**Non-PS**	**Total**
**Abnormal ioflupane [**^**123**^**I] images**^**a**^	26	0	26	20	2	22
**Normal ioflupane [**^**123**^**I] images**	2	20	22	1	21	22
**Total**	28	20	48	21	23	44
**Statistic**	**Result**	**Exact 95% CI**		**Result**	**Exact 95% CI**	***P*** **value/analysis method**^**b**^
**Sensitivity**^**c**^	0.9286	(0.7650, 0.9912)		0.9524	(0.7618, 0.9988)	1.0000
**Specificity**^**d**^	1.0000	(0.8316, 1.0000)		0.9130	(0.7196, 0.9893)	0.4906
**Accuracy**	0.9583	(0.8575, 0.9949)		0.9318	(0.8134, 0.9857)	0.6673
**Positive predictive value**	1.0000	(0.8677, 1.0000)		0.9091	(0.7084, 0.9888)	0.2048
**Negative predictive value**	0.9091	(0.7084, 0.9888)		0.9545	(0.7716, 0.9988)	1.0000

CI, confidence interval; CUPS, clinically uncertain parkinsonian syndrome; H&Y, Hoehn and Yahr; PS: parkinsonian syndrome. ^a^Abnormal imaging types 1, 2 and 3. ^b^*P* value is based on Fisher’s exact test. ^c^Sensitivity is equivalent to positive percent agreement. ^d^Specificity is equivalent to negative percent agreement.

### 3.3 Mini-mental state examination

At baseline, the majority of subjects had MMSE scores <29 (Table 3, Figure 1B). The number of subjects with normal and abnormal ioflupane [^123^I] imaging results was approximately equal for both groups, as were diagnoses of PS and non-PS at 1 year post scan. Sensitivity, specificity, accuracy, PPV, and NPV ranged between 94 and 95% for prediction of clinical diagnosis at 1 year, both in subjects with MMSE scores <29 and in subjects with MMSE scores ≥29. No noticeable differences were observed between low-scoring and high-scoring MMSE groups for any of the parameters.

**Table 3:** **Efficacy parameters for prediction of clinical diagnosis 1 year post scan: Mini-Mental State Examination (*n*** **= 92)**

**Subjects with CUPS**	**Clinical diagnosis at 1 year post-scan**					
	**Subgroup: MMSE <29**			**Subgroup: MMSE ≥29**		
	**PS**	**Non-PS**	**Total**	**PS**	**Non-PS**	**Total**
**Abnormal ioflupane [**^**123**^**I] images**^**a**^	19	1	20	27	1	28
**Normal ioflupane [**^**123**^**I] images**	1	16	17	2	25	27
**Total**	20	17	37	29	26	55
**Statistic**	**Result**	**Exact 95% CI**		**Result**	**Exact 95% CI**	***P*** **value/analysis method**^**b**^
**Sensitivity**^**c**^	0.9500	(0.7513, 0.9987)		0.9310	(0.7723, 0.9915)	1.0000
**Specificity**^**d**^	0.9412	(0.7131, 0.9985)		0.9615	(0.8036, 0.9990)	1.0000
**Accuracy**	0.9459	(0.8181, 0.9934)		0.9455	(0.8488, 0.9886)	1.0000
**Positive predictive value**	0.9500	(0.7513, 0.9987)		0.9643	(0.8165, 0.9991)	1.0000
**Negative predictive value**	0.9412	(0.7131, 0.9985)		0.9259	(0.7571, 0.9909)	1.0000

CI, confidence interval; CUPS, clinically uncertain parkinsonian syndrome; MMSE, Mini-Mental State Examination; PS, parkinsonian syndrome. ^a^Abnormal imaging types 1, 2 and 3.

^b^*P* value is based on Fisher’s exact test. ^c^Sensitivity is equivalent to positive percent agreement. ^d^Specificity is equivalent to negative percent agreement.

### 3.4 Subject’s age

At baseline, subjects were divided relatively equally between the younger group (<68 years of age) and the older group (≥68 years of age) (see Table 4, Figure 1C). The older group had slightly more subjects with abnormal ioflupane [^123^I] imaging results than the younger group. Likewise, diagnoses of PS and non-PS 1 year post dose were evenly divided for both younger and older age groups. The sensitivity, specificity, accuracy, PPV, and NPV of ioflupane [^123^I] imaging were all high (ranged between 91 and 100%) in both groups. Overall, no noticeable difference was observed between age groups for all tested parameters.

**Table 4:** **Efficacy parameters for prediction of clinical diagnosis 1 year post scan: subject’s baseline age (*n*** **= 92)**

**Subjects with CUPS**	**Clinical diagnosis at 1 year post-scan**					
	**Subgroup: <68 years of age**			**Subgroup: ≥68 years of age**		
	**PS**	**Non-PS**	**Total**	**PS**	**Non-PS**	**Total**
**Abnormal ioflupane [**^**123**^**I] images**^**a**^	22	0	22	24	2	26
**Normal ioflupane [**^**123**^**I] images**	2	20	22	1	21	22
**Total**	24	20	44	25	23	48
**Statistic**	**Result**	**Exact 95% CI**		**Result**	**Exact 95% CI**	***P*** **value/analysis method**^**b**^
**Sensitivity**^**c**^	0.9167	(0.7300, 0.9897)		0.9600	(0.7965, 0.9990)	0.6092
**Specificity**^**d**^	1.0000	(0.8316, 1.0000)		0.9130	(0.7196, 0.9893)	0.4906
**Accuracy**	0.9545	(0.8453, 0.9944)		0.9375	(0.8280, 0.9869)	1.0000
**Positive predictive value**	1.0000	(0.8456, 1.0000)		0.9231	(0.7487, 0.9905)	0.4929
**Negative predictive value**	0.9091	(0.7084, 0.9888)		0.9545	(0.7716, 0.9988)	1.0000

CI, confidence interval; CUPS, clinically uncertain parkinsonian syndrome; PS, parkinsonian syndrome. ^a^Abnormal imaging types 1, 2 and 3. ^b^*P* value is based on Fisher’s exact test. ^c^Sensitivity is equivalent to positive percent agreement. ^d^Specificity is equivalent to negative percent agreement.

### 3.5 Clinical syndrome predominance

Subjects with baseline motor signs with predominance of bradykinesia, rigidity, or postural instability had moderate (ranged between 67 and 89%) sensitivity, accuracy, and PPV of ioflupane [^123^I] imaging (see Table 5, Figure 1D). The specificity and NPV of ioflupane [^123^I] imaging were lower for the BRP dominant subgroup as compared with other clinical subgroups, both for tremor dominant, for BRP dominant versus tremor dominant, and for balanced subgroups (see Methods for definition of subgroups).

**Table 5:** **Efficacy parameters for prediction of ioflupane [**^**123**^**I] imaging, 1 year post scan: clinical syndrome predominance (*n*** **= 92)**

**Subjects with CUPS**	**Clinical diagnosis at 1 year post-scan**								
	**Subgroup A: BRP dominant**^**a**^			**Subgroup B: TD**^**b**^			**Subgroup C: balanced**^**c**^		
	**PS**	**Non-PS**	**Total**	**PS**	**Non-PS**	**Total**	**PS**	**Non-PS**	**Total**
**Abnormal ioflupane [**^**123**^**I] images**^**d**^	17	2	19	10	0	10	19	0	19
**Normal ioflupane [**^**123**^**I] images**	2	4	6	0	25	25	1	12	13
**Total**	19	6	25	10	25	35	20	12	32
**Statistic**	**Result**	**Exact 95% CI**	***P*** **value**^**e**^**(subgroup A vs. subgroup B)**	**Result**	**Exact 95% CI**	***P*** **value**^**e**^**(subgroup B vs. subgroup C)**	**Result**	**Exact 95% CI**	***P*** **value**^**e**^**(subgroup C vs. subgroup A)**
**Sensitivity**^**f**^	0.8947	(0.6686, 0.9870)	0.5320	1.0000	(0.6915, 1.0000)	1.0000	0.9500	(0.7513, 0.9987)	0.6050
**Specificity**^**g**^	0.6667	(0.2228, 0.9567)	0.0323	1.0000	(0.8628, 1.0000)	NE	1.0000	(0.7354, 1.0000)	0.0980
**Accuracy**	0.8400	(0.6392, 0.9546)	0.0259	1.0000	(0.9000, 1.0000)	0.4776	0.9688	(0.8378, 0.9992)	0.1575
**Positive predictive value**	0.8947	(0.6686, 0.9870)	0.5320	1.0000	(0.6915, 1.0000)	NE	1.0000	(0.8235, 1.0000)	0.4865
**Negative predictive value**	0.6667	(0.2228, 0.9567)	0.0323	1.0000	(0.8628, 1.0000)	0.3421	0.9231	0.6397, 0.9981)	0.2219

BRP, bradykinesia, rigidity, postural instability; CI, confidence interval; CUPS, clinically uncertain parkinsonian syndrome; NE, not estimable; PS: Parkinsonian syndrome; TD, tremor dominant. ^a^Maximal score of bradykinesia, rigidity, or postural instability > tremor score. ^b^Tremor score > maximal score of bradykinesia, rigidity, or postural instability. ^c^Maximal score of bradykinesia, rigidity, or postural instability = tremor score. ^d^Abnormal imaging types 1, 2 and 3. ^e^*P* value is based on Fisher’s exact test. ^f^Sensitivity is equivalent to positive percent agreement. ^g^Specificity is equivalent to negative percent agreement.

In contrast, all of the efficacy parameters of ioflupane [^123^I] imaging in subjects with tremor dominant and balanced motor signs ranged between 92 and 100%, showing specificity of 100% for the tremor dominant and balanced motor signs subgroups (see Table 5, Figure 1D).

In addition, we estimated the diagnostic effectiveness of baseline clinical diagnosis without availability of imaging results (Table 6) and compared it with the effectiveness of ioflupane [^123^I] imaging (Table 7). Although the range of observed specificity of baseline clinical diagnosis was 33 to 56% for the three subgroups, the difference in diagnostic performance between these groups for baseline clinical diagnosis was not significant for all tested parameters (Table 6).

**Table 6:** **Efficacy: baseline clinical diagnosis, prediction of clinical diagnosis 1 year post scan (clinical syndrome predominance,** ***n*** **= 92)**

**Subjects with CUPS**	**Clinical diagnosis at 1 year post-scan**								
	**Subgroup A: BRP dominant**^**a**^			**Subgroup B: TD**^**b**^			**Subgroup C: balanced**^**c**^		
**Baseline clinical diagnosis**	**PS**	**Non-PS**	**Total**	**PS**	**Non-PS**	**Total**	**PS**	**Non-PS**	**Total**
**Positive (PS)**	21	4	25	8	10	18	17	6	23
**Negative (non-PS)**	0	2	2	1	13	14	3	7	10
**Total**	21	6	27	9	23	32	20	13	33
**Statistic**	**Result**	**Exact 95% CI**	***P*** **value**^**d**^**(subgroup A vs. subgroup B)**	**Result**	**Exact 95% CI**	***P*** **value**^**d**^**(subgroup B vs. subgroup C)**	**Result**	**Exact 95% CI**	***P*** **value**^**d**^**(subgroup C vs. subgroup A)**
**Sensitivity**^**e**^	1.0000	(0.8389, 1.0000)	0.3000	0.8889	(0.5175, 0.9972)	1.0000	0.8500	(0.6211, 0.9679)	0.1069
**Specificity**^**f**^	0.3330	(0.0433, 0.7772)	0.3898	0.5652	(0.3449, 0.7681)	1.0000	0.5385	(0.2513, 0.8078)	0.6285
**Accuracy**	0.8519	(0.6627, 0.9581)	0.1335	0.6563	(0.4681, 0.8143)	0.5977	0.7273	(0.5448, 0.8670)	0.3480
**Positive predictive value**	0.8400	(0.6392, 0.9546)	0.0093	0.4444	(0.2153, 0.6924)	0.1055	0.7391	(0.5159, 0.8977)	0.4869
**Negative predictive value**	1.0000	(0.1581, 1.0000)	1.0000	0.9286	(0.6613, 0.9982)	0.2721	0.7000	(0.3475, 0.9333)	1.0000

Scores for motor signs were 1 for none, 2 for possible, and 3 for definite. Excluded were subjects with 1-year clinical diagnosis of inconclusive, subjects with missing ioflupane [^123^I] imaging result, or subjects with missing 1-year clinical diagnosis. BRP, bradykinesia, rigidity, postural instability; CI, confidence interval; CUPS, clinically uncertain parkinsonian syndrome; PS, Parkinsonian syndrome; TD, tremor dominant. ^a^Maximal score of bradykinesia, rigidity, or postural instability > tremor score. ^b^Tremor score > maximal score of bradykinesia, rigidity, or postural instability. ^c:^Maximal score of bradykinesia, rigidity, or postural instability = tremor score. ^d^*P* value is based on Fisher’s exact test. ^e^Sensitivity is equivalent to positive percent agreement. ^f^Specificity is equivalent to negative percent agreement.

**Table 7:** **Comparison: diagnostic performance of ioflupane [**^**123**^**I] imaging with baseline clinical diagnosis (clinical syndrome predominance,** ***n*** **= 92)**

**Subjects with CUPS: subgroup**	**Ioflupane [**^**123**^**I] imaging vs. 1-year clinical diagnosis as reference standard**			**Baseline clinical diagnosis vs. 1-year clinical diagnosis as reference standard**		***P*** **value**^**a**^
		**Result**	**Exact 95% CI**	**Result**	**Exact 95% CI**	
**Subgroup A: BRP dominant**^**b**^	**Sensitivity**^**c**^	0.8947	(0.6686, 0.9870)	1.0000	(0.8389, 1.0000)	0.2192
	**Specificity**^**d**^	0.6667	(0.2228, 0.9567)	0.3330	(0.0433, 0.7772)	0.5671
	**Accuracy**	0.8400	(0.6392, 0.9546)	0.8519	(0.6627, 0.9581)	1.0000
	**PPV**	0.8947	(0.6686, 0.9870)	0.8400	(0.6392, 0.9546)	0.6843
	**NPV**	0.6667	(0.2228, 0.9567)	1.0000	(0.1581, 1.0000)	1.0000
**Subgroup B: TD**^**e**^						
	**Sensitivity**^**c**^	1.0000	(0.6915, 1.0000)	0.8889	(0.5175, 0.9972)	0.4737
	**Specificity**^**d**^	1.0000	(0.8628, 1.0000)	0.5652	(0.3449, 0.7681)	<0.0001
	**Accuracy**	1.0000	(0.9000, 1.0000)	0.6563	(0.4681, 0.8143)	<0.0001
	**PPV**	1.0000	(0.6915, 1.0000)	0.4444	(0.2153, 0.6924)	0.0039
	**NPV**	1.0000	(0.8628, 1.0000)	0.9286	(0.6613, 0.9982)	0.3590
**Subgroup C: balanced**^**f**^						
	**Sensitivity**^**c**^	0.9500	(0.7513, 0.9987)	0.8500	(0.6211, 0.9679)	0.6050
	**Specificity**^**d**^	1.0000	(0.7354, 1.0000)	0.5385	(0.2513, 0.8078)	0.0149
	**Accuracy**	0.9688	(0.8378, 0.9992)	0.7273	(0.5448, 0.8670)	0.0129
	**PPV**	1.0000	(0.8235, 1.0000)	0.7391	(0.5159, 0.8977)	0.0244
	**NPV**	0.9231	0.6397, 0.9981)	0.7000	(0.3475, 0.9333)	0.2806

BRP, bradykinesia, rigidity, postural instability; CI, confidence interval; CUPS, clinically uncertain parkinsonian syndrome; NPV, negative predictive value; PPV, positive predictive values; TD, tremor dominant. ^a^*P* value is based on Fisher’s exact test. ^b^Maximal score of bradykinesia, rigidity, or postural instability > tremor score. ^c^Tremor score > maximal score of bradykinesia, rigidity, or postural instability. ^d^Maximal score of bradykinesia, rigidity, or postural instability = tremor score. ^e^Sensitivity is equivalent to positive percent agreement. ^f^Specificity is equivalent to negative percent agreement.

**Figure 2:**
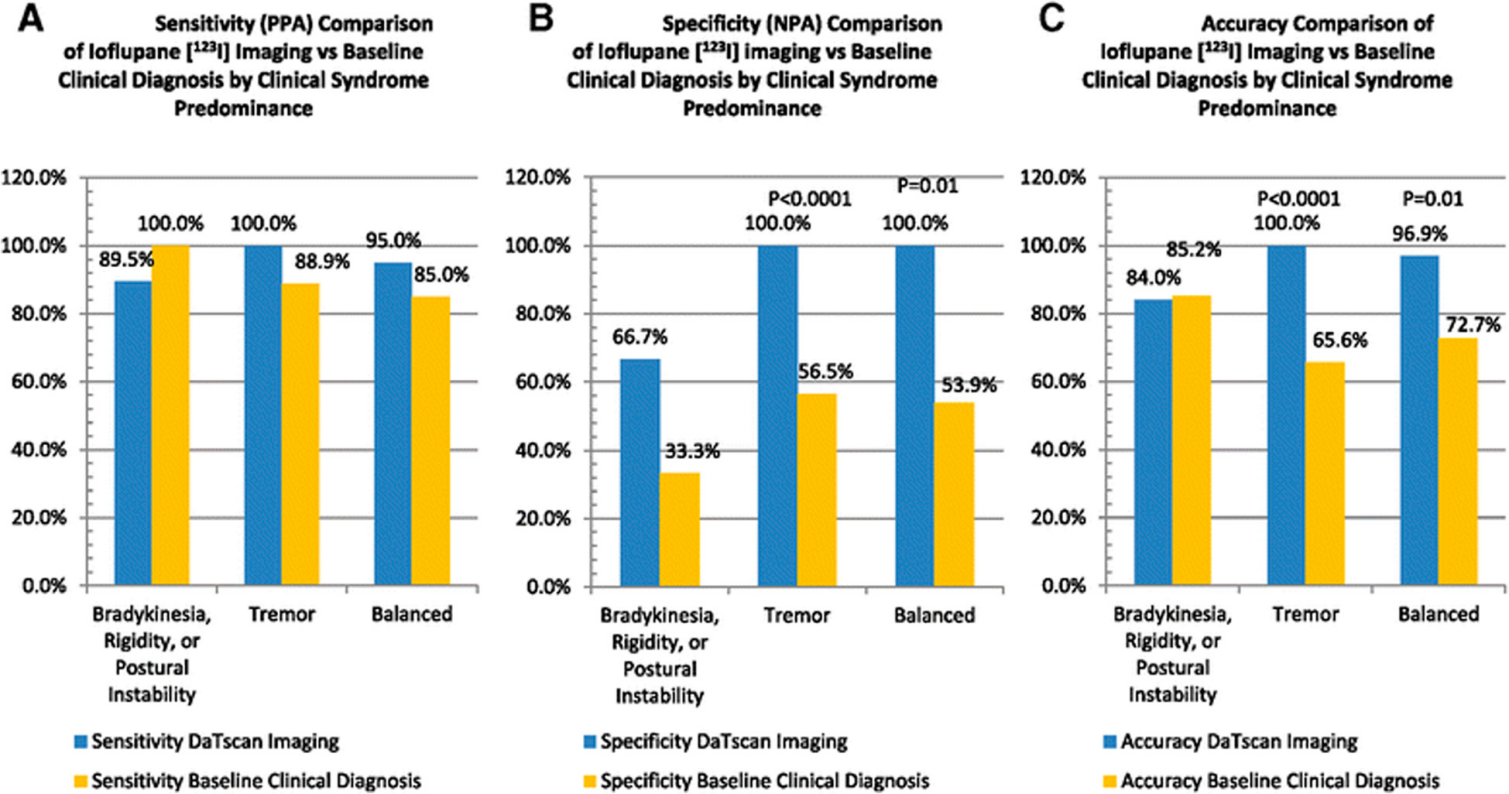
**Diagnostic performance of ioflupane [**^**123**^**I] imaging versus baseline clinical diagnosis. (A)** Sensitivity (PPA, positive percent agreement). **(B)** Specificity (NPA, negative percent agreement). **(C)** Accuracy.

For the BRP dominant subgroup, the difference between ioflupane [^123^I] imaging and baseline clinical diagnosis was not significant for all tested parameters (Table 7, Figure 2A).

Comparison of the specificity, PPV, and accuracy of ioflupane [^123^I] imaging with the same parameters for baseline clinical diagnosis (both compared with 1-year post-scan clinical diagnosis as reference standard) found significant differences for the tremor dominant and balanced subgroups (Table 7, Figure 2B,C). For the tremor dominant group, specificity, PPV, and accuracy were significantly higher for ioflupane [^123^I] imaging (all 100%) as compared with the baseline clinical diagnosis (ranged between 44 and 66%). For the balanced subgroup, specificity, PPV, and accuracy were significantly higher for ioflupane [^123^I] imaging (100%, 100%, and 97%) as compared with the baseline clinical diagnosis (54%, 73%, and 74%).

## 4 Discussion

### 4.1 Strengths and limitations of the study

The findings in this study are broadly in line with hypothetical predictive models and augment the scant literature examining the effects of age, clinical stage of PD and cognitive impairment on the utility of ioflupane [^123^I] imaging as an adjunct to clinical diagnosis. This study is the first robust statistical analysis of several potential influences on the efficacy of ioflupane [^123^I] imaging that measures their effect on image interpretation and supports the value of imaging in a real-world setting where clinicians are able to incorporate imaging results to assist them in making a diagnosis and subsequent treatment plan. The open-label setting is both a strength and a possible limitation of the study. Whilst the findings of the study indicate some utility of ioflupane [^123^I] in assisting diagnosis, one of the possible methodological limitations of this study is that information from the imaging scan could have influenced the clinician’s standard-of-truth diagnosis, causing a shift in the subsequent clinical management of the subject [1]. The onsite image evaluations by nuclear medicine physicians blinded to the clinical information were based on the findings of one qualified local reader per study site, and were not the consensus of a panel of multiple readers, a situation that was specifically designed to resemble clinical practice. At the time of the 1-year diagnosis, the general neurologists or movement disorder specialists had access to imaging information. The study was designed with a 1-year follow-up period post scan to be used as a reference standard, although the efficacy results between 90 and 100% had already been established in tremor and balanced subgroups, showing a potential ceiling effect for the test. It would be desirable to use a longer follow-up period for the BRP subgroup as a reference standard. Study subjects had clinical symptoms up to 5 years prior to enrolment in the study (mean 2.54 years), which in combination with the additional 1-year clinical follow-up and availability of imaging information should generally be sufficient for the final clinical diagnosis to be used as a reference standard in tremor and balanced subgroups [1]. This is the first study showing a difference for ioflupane [^123^I] imaging between different motor syndrome subgroups, including subjects with CUPS. Because of the modest study size and possible unequal distribution of subjects amongst motor syndromes, the small number of participants in the BRP dominant subgroup may not be sufficiently powered to adequately address real differences in imaging utility from other motor subgroups. Despite this, the overall accuracy of ioflupane [^123^I] imaging for the BRP dominant subgroup was still very good, although less than for the tremor dominant and balanced subgroups.

### 4.2 Discussion of findings

It has been established that SPECT imaging of presynaptic DaTs has utility in the premotor diagnosis of PD, as noted in analysis of cohorts presenting with REM sleep behavioral disorder or anosomia/hyposmia [8],[9]. Further, a large-scale audit of ioflupane [^123^I] imaging abnormality in patients at initial motor presentation of PD in a UK movement disorder service noted the majority of patients to be at stage 2 to 3 of ioflupane [^123^I] imaging abnormality [10],[11]. Taken together, these sets of data suggest the possibility that ioflupane [^123^I] imaging might be able to detect abnormality both at early (premotor) and later (motor) clinical presentation, although to date there has been no analysis of H&Y stage versus clinical utility of ioflupane [^123^I]. The findings in our study support this hypothetical notion of no significant effect of H&Y stage on diagnostic accuracy of ioflupane [^123^I].

Whether or not diagnostic utility of ioflupane [^123^I] imaging varies with stage of cognitive impairment in CUPS has also not been analyzed to date. The findings in our study of no effect of MMSE on accuracy of ioflupane [^123^I] imaging suggest the technique remains accurate in the context of cognitive impairment. Our analyses did not include subgrouping of mild, moderate, or severe dementia based on MMSE, which would have been useful, although data from the DLB Study Group would suggest the technique could be of diagnostic utility even in the presence of severe cognitive impairment [12],[13].

An average age-related decline of DaT availability of 5.5% across both genders per decade has been reported from work on normal healthy controls [14]. This reduction of DaT signal with age might affect diagnostic sensitivity of the technique if abnormal ioflupane [^123^I] imaging was to show subtle or minimal change between normal and abnormal cases. Previous reports correlating ioflupane [^123^I] image abnormality with first clinical presentation of PD [10],[11], however, suggest a reasonable correlation with work from Braak and colleagues [15] suggesting that ioflupane [^123^I] image abnormality at motor PD presentation is in line with an estimated 60 to 80% dopaminergic cell loss at motor presentation, and is in any case associated with asymmetrical striatal signal loss rather than the symmetrical loss due to ageing. In accord with the published literature, this would be expected to yield substantial ioflupane [^123^I] imaging abnormality, and the majority of patients in this study had grade 2 to 3 abnormality (moderate–severe) rather than grade 1 (mild). The mild age-related symmetric reduction in DaT binding with increased age would therefore probably not have a significant effect on diagnostic performance of this test, and the findings in our study are in keeping with this hypothesis.

There have been a number of reports on the pattern of ioflupane [^123^I] image abnormality seen in the motor subtype of PD analyzed, broadly tremor dominant versus akinetic-rigid phenotypes [16]. These studies did not analyze clinical utility of ioflupane [^123^I] imaging in making an accurate clinical diagnosis in the two phenotypes, although the areas of reduced dopaminergic projection differ between the two, with visible reduction to the dorsal putamen in akinetic-rigid patients and in the caudate nucleus in tremor dominant patients [16]. In general, the differential diagnosis of a tremulous parkinsonian presentation is more limited than that of an akinetic-rigid presentation. The former would include tremor dominant PD or an alternative benign tremulous condition where parkinsonian features might be noted. The latter has been termed benign indeterminate tremor by Deuschl and colleagues in the 1992 Movement Disorder Society tremor classification system [17]. A further differential to consider in these cases would be benign tremor with parkinsonism due to an alternative causation (for example, benign tremor on an age-related background of vascular parkinsonism) or a drug-induced tremor–parkinsonism causation (for example, sodium valproate exposure) [17],[18]. The differential clinical diagnosis of akinetic-rigid phenotypes (that is, BRP in the present analysis) is challenging and includes PD, multiple system atrophy, progressive supranuclear palsy, and Lewy body dementia, all of which would be expected to be associated with loss of dopamine transporters. In contrast, age-related changes – possibly vascular parkinsonism, medication-induced parkinsonism, normal pressure hydrocephalus, tardive parkinsonism secondary to neuroleptics, the Westphal variant of Huntington’s disease, Machado-Joseph disease (SCA-3), or manganese toxicity – are associated with more or less preserved presynaptic dopamine terminals, and with normal or less pathological ioflupane [^123^I] imaging scans [19]. Notably, a recent autopsy study including 16 patients with a diagnosis of probable PD at death reported at least seven pathologies not typically associated with dopamine terminal loss (3/12, no clear pathologic process; 1/12, hippocampal sclerosis; 1/12, vascular disease; 1/12, Alzheimer’s disease; 1/12, Alzheimer’s/vascular disease) [20]. Conceivably, although the present study was not controlled by postmortem data, similar clinical difficulties may account for the lower specificity and NPV in the BRP group in the present analysis. Given the wide range of differential diagnosis in an akinetic-rigid parkinsonian presentation, a longer duration may be needed to improve the reference standard of 1 year in less certain cases; but 1 year should be sufficient for the tremor dominant and balanced subtypes in generating an accurate long-term clinical diagnosis, although this proposition has not been formally tested. The data from this study are in keeping with these suppositions, showing a reduced diagnostic performance (specificity, NPV, and accuracy) in BRP presentations versus either tremulous or balanced motor presentation of disease. The diagnostic performance for ioflupane [^123^I] imaging for the tremor dominant and balanced subgroups was significantly better than for the baseline clinical diagnosis; for the BRP dominant subgroup, the difference did not reach statistical significance. The specificity of baseline clinical diagnosis for BRP was inferior to ioflupane [^123^I] imaging performance, supporting challenges for differential work-up in this motor syndrome, especially in subjects with CUPS. One strategy for improving the performance of the ioflupane [^123^I] imaging in the BRP subgroup could be a quantitative evaluation of SPECT images. Additionally, repeated ioflupane [^123^I] imaging scanning (for example, 12 or 24 months after baseline) could be helpful in those BRP patients with initial normal scans to determine progression of the disease.

According to our findings, the type of initial motor signs has an observable relationship with the predictability of performance of ioflupane [^123^I] imaging, with tremor dominant and balanced motor scores being more highly predictive of PS than bradykinesia, rigidity, or postural instability dominance. In our trial, the type of dominant motor signs, but not the H&Y stage, MMSE score, or age, impacted the diagnostic performance of ioflupane [^123^I] imaging. Ioflupane [^123^I] imaging had the strongest efficacy in tremor dominant and balanced cases of CUPS.

## 5 Conclusions

This study suggests that the diagnostic performance of ioflupane [^123^I] imaging in CUPS remains high at all stages of disease, including early stage, and across both age groups and cognitive state (MMSE). The strongest diagnostic performance of ioflupane [^123^I] imaging for clinical diagnosis of PS or non-PS was associated with subjects with tremor dominant and balanced motor scores rather than with BRP dominance. High diagnostic effectiveness of ioflupane [^123^I] imaging in diagnosis of CUPS was demonstrated by this study. Favorable performance of ioflupane [^123^I] imaging was observed relative to final clinical diagnosis at 1 year post scan.

## Abbreviations

BRP: bradykinesia, rigidity, postural instability

CUPS: clinically uncertain parkinsonian syndrome

DaT: dopamine transporter

H&Y: Hoehn and Yahr

MMSE: Mini-Mental State Examination

NPV: negative predictive value

PD: Parkinson’s disease

PPV: positive predictive value

PS: parkinsonian syndrome

SPECT: single-photon emission computed tomography

## Competing interests

CC is an employee of H2O Clinical LLC. IDG was an employee of GE Healthcare during the conduct of the study. The remaining authors declare that they have no competing interests.

## Authors’ contributions

NB participated in the research project (conception, organization, execution) and in manuscript preparation (review and critique). RAH participated in the research project (conception, organization, execution) and in manuscript preparation (review and critique). JS participated in the research project (conception, organization, execution) and in manuscript preparation (review and critique). AK participated in the research project (conception, organization, execution) and in manuscript preparation (review and critique). MP participated in the research project (conception, organization, execution) and in manuscript preparation (review and critique). CC was responsible for the statistical analysis (execution, review and critique) and participated in manuscript preparation (review and critique). CC takes responsibility for the integrity of the data and the accuracy of the data analysis. IDG participated in the research project (study team leadership, organization, execution, study medical director) and in manuscript preparation (review, critique, internal approval, and submission). IDG was a Global Medical Director of the Phase 4 Clinical Trial. All authors read and approved the final manuscript.

## Additional file

## Supplementary Material

Additional file 1: Table S1. Presenting the independent ethics committees and institutional review boards approving the clinical trial. Click here for file
