# Supplementary material for: Association between Hoehn and Yahr, Mini-Mental State Examination, age, and clinical syndrome predominance and diagnostic effectiveness of ioflupane I 123 injection (DaTSCAN™) in subjects with clinically uncertain parkinsonian syndromes
Source: Alzheimers Res Ther. 2014 Oct 8;6(5):67. doi: 10.1186/s13195-014-0067-0 (PMC4255542; doi:10.1186/s13195-014-0067-0)
Supplement: Additional file 1: Table S1. — Presenting the independent ethics committees and institutional review boards approving the clinical trial. [file s13195-014-0067-0-S1.docx]

**Supplemental Table S1. Independent ethics committees and institutional review boards approving the clinical trial**

| **Center Number** | **IEC/IRB Affiliation** |
| --- | --- |
| 001 | Ethik-Kommission des Landes Berlin  Landesamt für Gesundheit und Soziales Berlin  Geschäftsstelle der Ethikkommission des Landes Berlin  Sächsische Strasse 28  10707 Berlin  Germany |
| 002 | Ethik-Kommission der Ärztekammer Hamburg  Heinrich-Hertz-Straße 125  D-22083 Hamburg  Germany |
| 003 | Ethik-Kommission der Landesärztekammer Rheinland-Pfalz  Deutschhausplatz 3  55116 Mainz  Germany |
| 004 | Ethikommission für das Bundesland Salzburg  Amt der Salzburger Landesregierung  Sebastian-Stief-Gasse 2  A-5020 Salzburg  Austria |
| 005 | Southampton & South West Hampshire Research Ethics  Committee A  1st Floor, Regents Park Surgery  Park Street  Shirley  Southampton  SO16 4RJ |
| 006 | Northern & Yorkshire Research Ethics Committee  Room 002, TEDCO Business Centre  Viking Industrial Park  Rolling Mill Road  Jarrow  Tyne & Wear  NE32 3DT |
| 007 | Kantonale Ethikkommission Bern (KEK)  Murtenstraße 31  Postfach 56  CH-3010 Bern  Switzerland |
| 008, 018 | Regional Komite for Medisinsk Forskningsetikk  Helseregion Midt-Norge  Rådgiver Arild Hals  St Olavs Hospital  Det Medisinske Fakultet  Medisinsk Teknisk Forskningssenter  Kreftbygget 5.etg  N-7489 Trondheim  Norway |
| 009, 010, 011 | Comité de Protection des Personnes - Ouest V  Pavillon Clémenceau  Hôpital Pontchaillou  2 rue Henri Le Guilloux  35033 Rennes Cedex  France |
| 012 (Site not initiated) | Comitato Etico  Azienda Ospedaliera Villa Sofia - CTO  Viale Strasburgo n.233  90146 Palermo  Italy |
| 013 | Comitato Etico  Azienda Unità Sanitaria Locale n.5  Spezzino  Via XXIV Maggio n.139  19124 La Spezia  Italy |
| 014 | Comitato Etico dell'Az. Op. Santi Antonio e Biagio e Cesare  Arrigo de Alessandria  via Venezia 15  15100 Alessandria  Italy |
| 015 | Comité Ético de Investigatión Clínica  Hospital Universitario Ramón y Cajal  Planta -2 Derecha  Carretera del Colmenar Km 9.100  28034 Madrid  Spain |
| 016 | Comité Ético de Investigación Clínica  Hospital Universitario de Getafe  Ctra. De Toledo, Km 12.500  28905 Getafe (Madrid)  Spain |
| 017 | Comité Ético de Investigación Clínica de Galicia  Edificio Administrativo  C/San Lázaro s/n  15703 Santiago de Compostela - A Coruña  Spain |
| 019 | De Videnskabsetiske Komiteer for Region Hovedstaden  Regionsgården  Kongens Vænge 2  DK-3400 Hillerød  Denmark |
| 020 | Regionala Etikprövningsnämnden I Stockholm  Nobels väg 12 A, Solna  Box 289  SE-171 77 Stockholm  Sweden |
| 021 | South Glasgow and Clyde Research Ethics Committee  Ward 11, First Floor  Dykebar Hospital  Grahamston Road  Paisley  PA2 7DE |
| 023, 026 | Western Institutional Review Board (WIRB)  3535 Seventh Avenue  Olympia  WA 98502-5010  USA |
| 025 | Duke University Health System Institutional Review Board  Hock Plaza, 2424 Erwin Road  Suite 405, Campus Box 2712  Durham, NC 27705  USA |
| 027 | Sutter Health Central Area Institutional Review Committee  2801 Capitol Avenue, Suite 400  Sacramento, CA 95816  USA |
